# Post-Measles Complications and Mortality in Children under Five Years in Low- and Middle-Income Countries: A Narrative Review

**DOI:** 10.5334/aogh.5307

**Published:** 2026-10-26

**Authors:** Abhijeet V. Jadhav, Sankalp Ghadei, Sanket Sonawane, Pushkar Patil, Prashant Birajdar, Avinash Deoshatwar, Kunal Pise, Amol Nath, Shailesh Pawar, Naveen Kumar

**Affiliations:** 1 ICMR-National Institute of Virology, Pune, Maharashtra 411021, India; 2 ICMR- National Institute of Translational Virology and AIDS Research, Pune, Maharashtra 411026, India; 3 Armed Forces Medical College, Pune, Maharashtra 411040, India

**Keywords:** measles, post-measles complications, child health, vaccine-preventable diseases, low- and middle-income countries, immune amnesia

## Abstract

*Background:* Global measles vaccination programs have achieved significant reductions in measles-related mortality, with World Health Organization (WHO)-led initiatives expanding vaccine coverage to over 85% of children worldwide. Despite this progress, measles elimination remains elusive, undermined by anti-vaccine sentiment, armed conflicts, migration, refugee crises, and post-COVID-19 disruptions. No WHO region has achieved or sustained measles elimination status since the COVID-19 pandemic, and elimination remains challenging in resource-limited and conflict-affected contexts. A critical yet underappreciated consequence of this ongoing burden is the rise of post-measles complications (PMCs). Vaccine-preventable diseases and malnutrition constitute two critical global health priorities; PMCs represent an important intersection of these agendas that warrant greater attention within the global health framework.

*Objectives:* To review and synthesize current epidemiological evidence on PMCs, examine their pathogenesis, and understand the need for integrated post-measles care strategies in vulnerable populations.

*Methods:* This narrative review included all papers that published primary data on any of the PMCs in the last 55 years, identified through PubMed and Google Scholar.

*Findings:* Epidemiological data show that 30–40% of measles cases in low- and middle-income countries (LMICs) develop complications, including pneumonia, diarrhea, otitis media, and, less commonly, encephalitis and ocular disease. PMCs are frequently misattributed to independent illnesses because they often emerge after a delay following the acute infection. Compounding this, measles-induced immune amnesia renders affected individuals vulnerable to secondary infections for up to 2–3 years post-infection. In LMICs and conflict-affected settings, this prolonged immunological vulnerability intersects with pre-existing risk factors such as malnutrition, micronutrient deficiencies, and poor healthcare access, which amplify the consequences substantially.

*Conclusions:* As measles cases resurge globally, the associated burden of PMCs is expected to increase correspondingly. Where PMCs represent a pressing, underaddressed concern, integrated post-measles care strategies are urgently needed from a global health perspective, especially in resource-limited and conflict-affected settings.

## Introduction

Measles remains one of the leading causes of vaccine-preventable deaths in children. Approximately 107,500 and 95,000 people died from measles, mostly children under five, in 2023 and 2024, respectively. In 2024, 11 million cases were reported worldwide—nearly 800,000 more than in 2019, before the pandemic [1]. As per the World Health Organization (WHO), in 2024, over 20 million children did not receive their first measles vaccine, with only 84% and 76% global coverage for the first and second doses of the measles vaccine. Fifty-nine countries had significant or disruptive outbreaks in 2024, nearly three times more than in 2021, with a quarter occurring in countries that were previously free of measles [1–3].

The burden of measles falls disproportionately on low- and middle-income countries (LMICs), where weak health systems, inadequate vaccine coverage, and poor living conditions facilitate disease transmission and severe outcomes. The co-occurrence of measles infection and malnutrition results in a synergistic risk of mortality in these settings [4]. According to WHO estimates, approximately 149 million children under five are malnourished worldwide, with the highest burden in sub-Saharan Africa and South Asia [5]—the same regions experiencing the most measles deaths [6, 7]. In lower-income countries, 30–60 die out of 1000 measles-affected children compared to 1–2 in the USA [8]. One meta-analysis indicated a case fatality rate (CFR) of 3·03% in community-based settings for children less than 1 year and 1·63% for ages 1–4 years in 2019 [9]. Another study indicated a CFR of 6.1 among under-five children in the Democratic Republic of Congo [6]. Things may have worsened post-COVID and after recent armed conflicts.

There is a bidirectional relationship between measles and malnutrition, leading to a vicious cycle of disease and nutritional deterioration. Malnourished children are more susceptible to measles infection and experience more severe disease with higher mortality rates [10]. In return, measles infection exacerbates malnutrition through decreased food intake, impaired nutrient absorption, and increased metabolic demands. Furthermore, measles causes prolonged immune suppression lasting months to years after acute infection, which increases vulnerability to subsequent infections and mortality [4].

PMCs contribute to a major share of measles-related child mortality in LMICs, representing a major avoidable burden. The measles virus infects multiple organ systems, targeting epithelial, reticuloendothelial, and white blood cells—including monocytes, macrophages, and T lymphocytes. It uses SLAMF1 (CD150) on immune cells like T and B cells, macrophages, and dendritic cells for initial systemic spread, and Nectin-4 (PVRL4) on the basolateral surface of epithelial cells for shedding and transmission; vaccine and lab-adapted strains also bind the ubiquitous CD46 receptor [11–13]. Common complications include pneumonia, diarrhea, conjunctivitis, and secondary bacterial infections, with malnourished children at particularly high risk [14]. In the post-measles phase, vitamin A deficiency—affecting around 190 million children globally—exacerbates outcomes through its bidirectional link with complications, raising CFRs [15, 16].

Global measles deaths declined by 73% between 2000 and 2018 [17], yet elimination remains elusive in many LMICs, especially the WHO African Region, amid declining vaccination coverage, conflicts, vaccine hesitancy, and COVID-19 disruptions to immunization. Under-five children face higher risk from immature immune systems, high nutritional needs, and feeding vulnerabilities; severe measles combined with malnutrition may impair long-term neurodevelopment, educational attainment, and future productivity [18, 19].

This narrative review examines the full spectrum of interactions between measles infection, malnutrition, and PMCs using recent literature and epidemiological evidence in the context of LMICs. Understanding these interactions is essential for developing targeted interventions. Addressing these interactions requires a global health systems lens that integrates nutrition, immunization, and infectious disease programs, particularly within the context of universal health coverage and equity-focused primary care in LMICs.

### Challenges in measles elimination

Measles, with a basic reproduction number (R_0_) of 12–18 [20], requires ≥95% vaccination coverage for herd immunity [21, 22]. The Global Vaccine Action Plan (2011–2020) targeted measles elimination in five WHO regions by 2020 [23]. WHO strategies emphasize two-dose measles-containing vaccine (MCV) coverage ≥95%, strengthened surveillance, and supplementary immunization activities. Between 2000 and 2016, vaccination prevented an estimated 20.4 million deaths through the Measles & Rubella Initiative [24]. Post-COVID, some countries, such as Colombia, have successfully addressed immunity gaps [25]. However, despite progress, elimination remains elusive in LMICs. The proportion of countries with measles incidence >5 cases per million rose from 38% (2000) to 70% (2016), but progress has since stalled [26]. Global MCV1 coverage has remained in the range of 83–86% since 2010, and MCV2 is approximately 76% [2]. Poor cold chains, delayed diagnosis, and inadequate case management continue to sustain transmission. Subnational coverage gaps create susceptible reservoirs [27]. Infants aged 6–12 months face the highest risk of measles infection due to waning maternally derived antibodies, with most infants lacking passive immunity by 9–12 months [28]. Cyclical epidemics occur every 2–5 years in high-fertility LMICs [29, 30]. Countries including Albania, Cambodia, Venezuela, Germany, the United Kingdom, and Uzbekistan have lost elimination status due to population movements, civil unrest, declining coverage, or anti-vaccine movements [31–34], demonstrating that sustained high coverage and surveillance are essential. Susceptible populations remain at elevated risk of PMCs, and this risk will persist until measles elimination is achieved.

### Pockets of vulnerability and the maintenance of circulation

The drivers of measles vulnerability are closely associated with marginalized or disadvantaged populations. Poverty underlies multiple risk factors, including malnutrition, limited healthcare access, crowded living conditions, low vaccination rates, and low health literacy. Children from the poorest households face substantially elevated risks of both exposure to measles and severe outcomes when infected [35].

Geographic pockets of vulnerability frequently coincide with socioeconomic disadvantage, creating clusters where multiple risk factors converge. Migrant populations, internally displaced people, refugees, and marginalized ethnic or religious minorities frequently have reduced access to routine health services, including vaccination programs [36]. In December 2025, there were 239 million people who needed humanitarian assistance across the globe [7]. After this period, there was an increase in people facing war or a similar situation in early 2026, most of whom are from LMICs. Language barriers, discrimination, lack of documentation, and mistrust of health authorities further impede engagement with immunization services in these populations. MCV1 coverage in low-income countries is as low as 66% [3]. Such risk factors interact synergistically to amplify vulnerability to measles and its outcomes, explaining persistent inequities in measles burden and the challenges of achieving elimination in many LMICs [37, 38].

## Methods

This narrative review was conducted to synthesize and critically evaluate the published literature on post-measles complications (PMCs), associated mortality, and the pathophysiological interactions between measles, malnutrition, and immune suppression. The focus is on children under 5 years in LMICs. A narrative review design was selected because the primary objective was to provide a comprehensive, integrated overview of a broad and heterogeneous body of evidence spanning multiple complication types, biological mechanisms, and epidemiological settings, rather than to answer a single focused research question through systematic quantitative synthesis.

Literature searches were conducted in PubMed and Google Scholar (1971–2026) for primary studies evaluating PMCs. Search strings combined measles-related terms (“measles” and “rubeola”) with specific complications, including pneumonia, otitis media (OM), diarrhea, encephalitis/acute disseminated encephalomyelitis (ADEM), subacute sclerosing panencephalitis (SSPE), keratitis/blindness, malnutrition, and immune amnesia or suppression. Eligible studies reported primary epidemiological, clinical, or laboratory findings regardless of study design, geographic setting, or age group. Narrative reviews, editorials, and case reports lacking extractable primary data were excluded from incidence summaries. Titles, abstracts, and full texts were screened by [SG, SS, PP, and PB], with discrepancies resolved by consensus [AJ, SG, SS, ARD, KP, and AN].

Because some included studies were retrospective, cross-sectional, or hospital case series from heterogeneous settings, reported findings predominantly reflect epidemiological associations rather than definitive causal links. In keeping with the narrative design of this review, no formal risk-of-bias assessment was conducted.

## Results

### Post-measles complications: An understudied dimension

Current guidelines of most LMICs do not focus on PMCs or post-measles care and follow-up. While acute measles mortality has received substantial attention in public health discourse, the full spectrum of PMCs and their contribution to child morbidity and mortality warrants greater scrutiny. The prolonged immunosuppression following measles infection increases vulnerability to a wide range of pathogens for months to years after apparent recovery from acute infection [39, 40]. Table 1 summarizes most of the PMCs with their reported incidence rates along with the primary data references.

**Table 1:** Summary of studies reporting post-measles complications—Study characteristics and reported incidence.

COMPLICATION CATEGORY	AUTHOR (YEAR) [REF]	STUDY DESIGN	COUNTRY	SETTING	POPULATION	SAMPLE SIZE (N)	COMPLICATION	INCIDENCE / PERCENTAGE
**Respiratory complications**	Ammar et al. (2026) [41]	Cross-sectional study	Iraq	Pediatric hospital	Children with confirmed measles	200	Otitis Media (OM)	45%
	Ilyas et al. (2020) [42]	Retrospective descriptive study	Pakistan	Tertiary care hospital, Peshawar	Children with measles (early childhood)	100	Otitis Media (OM)	6%
	Tolera et al. (2025) [43]	Cross-sectional study	Ethiopia	Jimma University Medical Center	Children hospitalized with suspected measles	250	Pneumonia	82%
**Neurological complications**	Ali Deeba et al. (2020) [44]	Review paper with literature-derived estimate	LMICs	Hospital (case in Belgium)	Non-vaccinated patient with measles	NA	Acute Disseminated Encephalomyelitis (ADEM)	~1 per 1,000 measles cases
	Garg and Pandey (2025) [45]	Narrative literature review	LMICs (multi-country)	Community/hospital (pooled)	Measles-affected general population	NA	Subacute Sclerosing Panencephalitis (SSPE)	27.9 per 100,000 measles cases
**Ocular complications**	Sauter (1976) [46]	Observational study	Kenya	Hospital	Measles patients	267	Measles-associated Keratoconjunctivitis	87.6% (234/267)
	Foster and Sommer (1987) [47]	Prospective cohort study	Tanzania	Hospital/community	Children with measles	130	Corneal Ulceration	37%
	Sandford-Smith and Whittle (1979) [48]	Retrospective study	Nigeria	Hospital	Children with measles	89	Corneal Ulceration	42%
	Animashaun (2024) [49]	Retrospective observational study	Nigeria	Hospital	Children with measles	2772	Measles-associated Blindness (ocular complications)	1% (29/2,772)
**CONFLICT-AFFECTED COUNTRIES**								
**Complications**	Masters et al. (2023) [50]	Descriptive epidemiological outbreak investigation	USA	US military resettlement bases (Afghan evacuees)	Afghan evacuees under humanitarian interventions	47	Diarrhea	53%
							Otitis Media	6%
							Pneumonia	6%

*Abbreviations:* NA = Not Applicable; ADEM = Acute Disseminated Encephalomyelitis; SSPE = Subacute Sclerosing Panencephalitis; LMICs = Low- and Middle-Income Countries.

*Note:* Because included studies vary markedly in design, study population, setting, sample size, and case definitions, reported percentages represent heterogeneous data and cannot be directly compared across studies or pooled as uniform incidence estimates.

PMCs are defined here as clinical sequelae arising during or after acute measles that share a temporal and causal relationship with the infection. Acute complications (e.g., pneumonia, diarrhea, OM, and conjunctivitis) arise during or within 3 weeks of illness via direct viral cytopathic and early secondary bacterial superinfection. Subacute complications (e.g., ADEM) emerge after 3 weeks to 3 months after infection and commonly include immune-mediated mechanisms. Finally, long-term sequelae (notably SSPE and outcomes of immune amnesia) present after 3 months.

PMCs can affect up to 40% of patients, with particularly high rates among young children and those with malnutrition [51]. The occurrence of PMCs also depends on the pre-existing health status of children and the strength of the public health system. Marked variability in complication frequencies across Table 1 stems from differences in cohort age structure, baseline nutritional status, healthcare access, study area, timelines, and clinical setting. Studies conducted in tertiary hospital settings are likely to over-represent more severe presentations relative to community-based estimates. Hence, these figures cannot be directly used to compare PMC incidence rates but give a broad understanding of their occurrence.

Measles-associated mortality often occurs days to weeks after acute infection, mediated by secondary infections and nutritional deterioration rather than direct viral effects alone. This delayed mortality may be underestimated in surveillance systems that focus on deaths during the acute illness. Furthermore, long-term sequelae, including impaired neurodevelopment and reduced educational attainment, may extend the societal impact of measles well beyond immediate health outcomes [52].

### Immune compromise and post-measles infections

The measles virus exhibits a strong tropism for immune cells that express the receptor SLAM/CD150, including memory T and B-lymphocytes, dendritic cells, and subsets of monocytes/macrophages [53–55]. After initial replication in the respiratory epithelium and regional lymphoid tissue, infected lymphocytes disseminate the virus systemically, seeding lymphoid organs. During the acute rash phase, lymphocyte depletion can be profound, falling to as low as one-seventh to one-tenth of normal values [53, 55]. Studies also indicate that there is immune-mediated clearance of MV-infected CD45RA (-) memory T-lymphocytes and follicular B-lymphocytes, causing immune amnesia [56]. Also, there may be a compromised function of dendritic cells and T-helper 1 cell responses [57]. This systemic lymphopenia affects both naive and memory compartments and is accompanied by functional impairment of surviving T cells and antigen-presenting cells, which together drive the characteristic immunosuppression of acute measles [58].

Beyond this transient lymphopenia, measles causes a deeper and longer-lasting disruption of immunological memory that has been described as “immune amnesia” [59, 60]. Studies show that measles infection depletes pre-existing memory B cells and contracts the diversity of the antibody repertoire directed against previously encountered pathogens, effectively erasing established humoral memory [61]. Measles can eliminate 11–73% of the host’s pre-existing functional antibody repertoire, confirming that it directly depletes immunological memory rather than merely causing transient dysfunction [59]. Reconstitution of the B-cell pool after recovery occurs primarily through new naïve B-cell responses, meaning that the host must re-establish immune protection against pathogens to which it was already immunologically primed [59, 61].

Transient immunosuppression and immune amnesia are distinct immunological consequences of measles virus tropism for Signaling Lymphocytic Activation Molecule SLAM-expressing cells [53]. While peripheral lymphocyte counts typically normalize within a week, functional immunosuppression endures for weeks to months, driving immediate vulnerability to secondary bacterial complications like pneumonia and OM [53, 58]. In contrast, immune amnesia erodes pre-existing humoral and cellular memory for 2–3 years [59, 60]. This prolonged deficit leaves children vulnerable to a wide array of previously encountered pathogens long after the resolution of acute lymphopenia [59, 61].

Epidemiological data show that excess mortality from infectious diseases remains elevated for 2–3 years after measles outbreaks, consistent with long-term impairment of immunological memory [59, 60]. This state creates a permissive environment for bacterial invasion through several synergistic mechanisms. First, depletion and functional impairment of CD4+ and CD8+ T cells compromise macrophage activation, cytokine signaling, and cytotoxic responses, weakening control of both intracellular and extracellular bacterial pathogens [62]. Second, loss of antigen-specific memory B cells and erosion of pre-existing antibody repertoires reduce opsonizing and neutralizing antibodies against common bacterial agents, impairing clearance of common but serious pathogens that frequently cause post-measles pneumonia, OM, and sepsis [53, 63]. Third, direct measles-mediated damage to respiratory epithelium and mucosal barriers facilitates bacterial colonization, overgrowth, and translocation [47, 59]. Together, these immunological effects explain how measles infection chronically predisposes children to severe secondary bacterial infections and associated complications [53, 59–61].

#### Otitis media (middle ear infections)

OM is one of the most common PMCs, affecting 7–9% of cases globally [63]. It is mostly non-fatal but primarily affects children under 5 years. The main mechanism is post-measles immunosuppression and immune amnesia, which enables opportunistic secondary bacterial infections with *Streptococcus pneumoniae* or *Haemophilus influenzae* [53]. Additionally, the measles virus directly damages and inflames the respiratory epithelium lining the Eustachian tube; the resulting blockage, combined with immune deficiency, substantially increases the risk of bacterial colonization and suppurative OM.

#### Pneumonia and other pyogenic infections (skin, sepsis)

Pneumonia is the most common cause of measles-related death [14], particularly in LMICs where hospital CFRs remain high. Beyond the immunosuppressive mechanisms described earlier, the measles virus exerts direct cytopathic effects on the respiratory epithelium, leading to denudation of ciliated cells and impairment of mucociliary clearance [64]. This disruption of the primary mechanical defense barrier facilitates translocation of opportunistic bacterial pathogens—predominantly *S. pneumoniae* and *H. influenzae*—into the compromised pulmonary parenchyma, culminating in severe secondary bacterial pneumonia [52].

Beyond pulmonary complications, the immunosuppressed state predisposes patients to pyogenic infections. Deep skin and soft-tissue infections, including abscesses and noma (cancrum oris—a devastating orofacial gangrene affecting malnourished children) following measles, can rapidly progress to sepsis [65, 66]. Sepsis is a significant contributor to catastrophic outcomes in pediatric measles infections, emphasizing the critical importance of vigilant monitoring and prompt antibiotic intervention when secondary infection is suspected.

### Malnutrition and nutritional deficiencies

Measles and malnutrition have a lethal reciprocal interaction that results in a vicious cycle of illness and nutritional decline [63]. This bidirectional relationship is well-documented: malnutrition predisposes children to severe measles, while measles infection is itself a significant contributor to the development and exacerbation of malnutrition [67].

Due to impaired T-cell activity and mucosal immunity, malnourished children are particularly vulnerable to severe measles infection. Severe protein malnutrition causes thymic atrophy with reduced cell numbers and subsequently underdeveloped peripheral lymphoid organs, leading to long-lasting immune defects characterized by leukopenia, a decreased CD4:CD8 ratio, and increased numbers of immature T cells [68]. These immunological impairments render malnourished children significantly more susceptible to measles and its complications. Malnutrition is a primary contributor to death in 45% of fatal measles cases [69].

Measles worsens malnutrition through multiple pathophysiological mechanisms [63]:

- **Decreased food intake (anorexia):** Anorexia, nausea, and vomiting may be associated with electrolyte loss, imbalance, and dehydration during measles infection, directly compromising nutritional status during the acute phase of illness.
- **Malabsorption and gastrointestinal dysfunction:** Acute measles infection causes significant gut malabsorption, particularly in the proximal small intestine. The measles virus can directly damage the intestinal lining, impair nutrient absorption, and cause substantial protein loss. Children who are undernourished and develop measles are especially at risk of severe malnutrition, including kwashiorkor, due to higher protein and energy needs during illness, reduced food intake, and ongoing gut losses [4]. Protein-losing enteropathy is typically more severe and prolonged after measles, particularly when complicated by co-infections such as Shigella and enterotoxigenic *Escherichia coli* [70].
- **Elevated metabolic demands:** The febrile response and immune activation during measles significantly increase metabolic requirements [67]. A study in Nigeria found that severe metabolic demands during acute measles further deteriorated the condition of malnourished children, leading to weight loss, wasting, and reduced serum levels of essential amino acids [66].
- **Diarrheal disease:** Measles-associated diarrhea contributes significantly to malnutrition through reduction in food intake, decreased nutrient absorption, and increased catabolism of nutrient reserves. Studies measuring fecal α-1 antitrypsin found that 87% of patients with shigellosis, 63% with enterotoxigenic *E. coli*, and 42% with rotavirus diarrhea experienced significant protein loss [71].

#### Vitamin A depletion and its consequences

Vitamin A is crucial for immune function and the maintenance of epithelial integrity. Acute measles triggers vitamin A deficiency by depleting stores and increasing utilization; hyporetinemia (low serum vitamin A) may occur even when hepatic stores are adequate, because vitamin A is not mobilized rapidly enough to meet increased demand during infection [72, 73]. As a result, serum vitamin A levels in well-nourished children with measles can fall to lower levels than those of malnourished children without measles [74]. This drop has serious consequences: vitamin A deficiency is associated with more severe measles, longer recovery times, and greater risk of xerophthalmia, corneal ulcers, corneal perforation, and permanent blindness. CFRs are substantially higher in young vitamin A-deficient children, particularly in LMICs.

Clinical trials demonstrate that vitamin A supplementation reduces overall mortality and pneumonia-specific mortality in children with measles. Its benefits are attributable to distinct mechanisms from general immunomodulation: specifically, vitamin A restores mucosal integrity after viral infection, improves antibody responses to bacterial antigens, and supports neutrophil function recovery, thereby protecting against opportunistic bacterial infections [73]. Although vitamin A does not appear to improve measles-specific cellular immune function (cytotoxic T-lymphocyte [CTL] or delayed-type hypersensitivity [DTH] responses), it does augment the serum IgG response and plays an important role in mucosal defense—effects that are mechanistically distinct from, and complementary to, the lymphocyte-mediated immune amnesia described earlier.

### Neurological complications, including encephalitis

ADEM, occurring in approximately 1 in 1000 measles cases, is the most common neurological consequence linked to measles infection [44]. ADEM is an autoimmune disorder in which the immune system inadvertently targets the central nervous system’s (CNS) myelin sheath, causing acute demyelination [17]. Onset typically occurs 1–2 weeks after the acute rash and may present with encephalopathy, focal neurological deficits, seizures, and ataxia. In ADEM, no single confirmatory biomarker exists; diagnosis rests on a monophasic clinical presentation of acute encephalopathy with polyfocal neurological deficits, supported by MRI evidence of widespread, asymmetric, poorly demarcated white matter lesions on T2/FLAIR sequences. Cerebrospinal fluid (CSF) findings—mild pleocytosis and elevated protein—are non-specific and must be interpreted alongside neuroimaging, as normal CSF does not exclude the diagnosis [75, 76]. Management is primarily supportive, with corticosteroids used in severe cases, although outcomes are variable [72].

SSPE is a rare but universally fatal late complication of measles, caused by persistent CNS infection with a defective, mutated measles virus [77]. Following acute measles, viral particles harboring mutations in the matrix (M) protein accumulate in neurons and glial cells, evading immune clearance and establishing a slowly progressive, destructive encephalitis [45]. Spread within the CNS occurs trans-synaptically rather than via the bloodstream, explaining the insidious onset and the characteristically absent measles virus in CSF on standard testing.

SSPE progresses through four recognizable clinical stages: (I) subtle behavioral changes, personality alteration, and cognitive decline; (II) myoclonic jerks, deteriorating intellectual function, and characteristic periodic EEG complexes (Radermecker complexes); (III) rigidity, autonomic instability, and loss of purposeful movement; and (IV) a persistent vegetative state preceding death. The median latency from primary measles infection to clinical onset is 5–10 years, meaning that children infected in infancy may not present until school age or early adolescence [78]. Median survival from symptom onset is 1–3 years. Because standard CSF analysis does not detect measles virus, diagnosis relies on markedly elevated CSF measles antibody titers and characteristic EEG findings [45].

The risk of SSPE is strongly age-dependent, with estimates in LMICs reaching 27.9 per 100,000 measles cases among children infected before the age of two [45]—far exceeding historical population-level estimates of 1 per 100,000.

### Ocular complications

Measles-associated conjunctivitis is a prodromal phase symptom characterized by red, watery eyes, weeping, and mild photophobia [79]. It affects nearly all measles patients and is often a minor, self-limiting inflammation that fades as the systemic infection progresses [80]. It serves as an important early diagnostic signal alongside coryza and cough [79]. Although the primary inflammation is viral, subsequent bacterial superinfection is possible, necessitating watchful eye care in children—particularly in high-risk groups [81].

Keratitis, or corneal inflammation, is a more dangerous ocular consequence that can cause permanent vision impairment [80]. Measles keratitis can progress from superficial punctate erosions to profound corneal ulcers through direct viral destruction and secondary bacterial invasion [81]. Malnutrition, particularly vitamin A deficiency, significantly increases risk, predisposing to severe keratomalacia (corneal melting) [82]. Prompt diagnosis and high-dose vitamin A supplementation are critical to prevent corneal scarring and blindness.

Measles remains a leading cause of childhood blindness, accounting for an estimated 15,000–60,000 new cases each year, the vast majority in low-income countries. Visual loss is frequently caused by severe keratitis and corneal scarring (adherent leucoma) [82]. The combination of measles infection with pre-existing vitamin A deficiency has a synergistic and destructive effect, accelerating corneal damage [77, 82]. In Tanzania, 37% of corneal ulcers in a hospital cohort of children were associated with recent measles infection [47]; in Nigeria, at least 42% of corneal scars in a pediatric survey were attributed to post-measles ulceration [48].

Measles retinopathy—direct inflammation or injury to retinal tissue—can cause temporary or permanent vision loss, sometimes years after the initial infection [81]. Optic neuritis presents with acute eye pain and significant, usually transient, color blindness, and visual acuity impairment [80, 81]. Both conditions are rare but serious, necessitating prompt specialist evaluation.

### Other rare complications

Beyond the common PMCs described above, several rare manifestations have been documented. Gastrointestinal involvement includes hepatitis with elevated transaminases and appendicitis with characteristic measles giant cells on histopathology. Neurological complications extend beyond ADEM to include viral meningitis and post-infectious encephalomyelitis with immune-mediated demyelination. The pantropic nature of the measles virus enables multisystem involvement, particularly among young children, adults over 20 years, pregnant women, and immunocompromised individuals [83].

## Discussion

PMCs represent a disproportionate concern for LMICs, where overlapping vulnerabilities such as malnutrition, vitamin A deficiency, limited healthcare access, and crowded living conditions may amplify both their frequency and severity. While most high-income countries have achieved or are approaching measles elimination, the case burden remains concentrated in sub-Saharan Africa, South Asia, and parts of the Eastern Mediterranean [1, 37, 38]. These are precisely the regions where post-measles surveillance and care infrastructure is relatively less developed, compounding the risk to individual children and to the communities.

In conflict settings, the risk of severe PMCs is compounded by the near-total absence of the post-measles follow-up care that might otherwise mitigate secondary complications. The malnutrition burden prevalent among conflict-displaced and refugee populations further synergizes with measles-induced immune suppression, amplifying vulnerability to the full spectrum of PMCs. Children who survive acute measles in these environments may face PMCs without any prospect of structured follow-up, nutritional rehabilitation, pneumococcal vaccination, or other feasible interventions. Furthermore, the high prevalence of acute malnutrition among conflict-affected or displaced populations synergizes with measles-induced immune suppression, creating conditions of elevated all-cause infectious mortality for a couple of years, as documented in multicountry LMIC studies [59] and a study from the Netherlands [60]. The PMCs are likely to be substantially prolonged or further amplified. The long-term excess mortality attributable to measles-induced immune amnesia, already documented across LMIC settings [39], may therefore be even greater in conflict-affected contexts than existing epidemiological data suggest.

Unvaccinated children consistently bear a disproportionate share of PMCs, even where breakthrough infections constitute most reported cases. In Iraq, despite 71% of confirmed measles occurring in children with at least one prior vaccine dose, unvaccinated children had 3.2-fold higher odds of developing pneumonia [41]. Similarly, hospital-based data from Pakistan showed that PMCs predominated in unvaccinated cohorts [42]. These findings indicate that while breakthrough cases frequently occur in settings with cold-chain deficits, incomplete dosing, or waning immunity, vaccination may retain some protection against severe complications. There may be a confounder that vaccinated individuals are better off and may have better access to healthcare. This aspect needs more conclusive evidence.

Despite the breadth of literature on individual PMC types, significant evidence gaps remain. There is a critical absence of prospective longitudinal cohort studies tracking children beyond the acute measles episode in LMIC settings. Population-based neurological complications data from high-burden countries are sparse; long-term neurodevelopmental outcomes in measles survivors from LMICs are poorly characterized. The standardized case definitions for PMCs may enable comparison and burden estimation in LMICs. Addressing these gaps should be a priority for global health research.

Translating epidemiological insights into program action is an urgent need, especially in destabilized communities. Improvements in measles case-based surveillance across many LMICs mean that most measles patients are now identifiable at the point of care. This can create an opportunity to link these cases to existing programs for nutritional rehabilitation; pneumococcal vaccine given the elevated post-measles risk of bacterial pneumonia and OM [52, 63]; and integration with existing deworming programs, given the prolonged immune vulnerability of the post-measles period. Structured health monitoring for 12–24 months post-infection, aligned with the known duration of immune amnesia, would simultaneously reduce PMC-attributable morbidity and generate the longitudinal evidence currently lacking [59, 60]. The WHO Integrated Management of Childhood Illness (IMCI) framework provides an existing operational model for integrating post-measles care protocols at the primary healthcare level across LMICs [84].

## Conclusion

PMCs spanning respiratory, gastrointestinal, neurological, and ocular sequelae alongside measles-induced immune amnesia represent a major, neglected burden in LMICs. The disease burden extends well beyond the acute phase, leaving children vulnerable to secondary infections during an extended period of immunosuppression. However, existing evidence remains heterogeneous and sparse, particularly regarding post-acute outcomes.

Critical knowledge gaps include a scarcity of prospective longitudinal cohorts, unstandardized case definitions, limited population-based data on neurological sequelae, and insufficient evidence stratifying risk by vaccination status. Addressing these gaps demands dedicated longitudinal and implementation research in high-burden settings. Concurrently, measles elimination strategies must incorporate structured post-infection care to support nutritional recovery, facilitate early complication detection, and sustain clinical follow-up. Established child health frameworks, notably the WHO IMCI, offer an immediate operational platform to integrate post-measles monitoring into routine primary care protecting vulnerable children while generating essential longitudinal evidence.

## Data Availability

This is a narrative review; no new primary data were generated or analyzed. All data discussed are drawn from previously published, publicly available sources, which are cited in the reference list. If requested, these extracted data can be shared.

## Co-Authors’ Sign-Off

All co-authors have read the final version of this manuscript and approved it for submission to the journal.

## Third-Party/Restricted Content Permissions

This manuscript does not reproduce any figures, tables, images, or substantial text from copyrighted third-party sources; all data presented are original syntheses of previously published findings, appropriately cited. No permissions were therefore required.
